# Comment on ‘Change in Physical Activity and Its Association With Decline in Kidney Function: A UK Biobank‐Based Cohort Study’ by Liu et al.

**DOI:** 10.1002/jcsm.13654

**Published:** 2024-11-20

**Authors:** Guoying Wang, Wenbo Shi, Zhijun Xin, Xiaoming Zhou

**Affiliations:** ^1^ Department of Critical Care Medicine The Second People's Hospital of Dongying Dongying China; ^2^ Department of Medical Oncology Ruijin‐Hainan Hospital, Shanghai Jiao Tong University School of Medicine Qionghai China; ^3^ Department of Surgical Anesthesiology The Affiliated Yantai Stomatological Hospital, Binzhou Medical University Yantai China; ^4^ Department of Research Shandong Provincial Hospital Affiliated to Shandong First Medical University Jinan China; ^5^ Department of Pharmacy Dongying People's Hospital Dongying China

Dear Editor,

We read with great interest the article by Liu Q et al. [1] examining the association between changes in physical activity and kidney function in the general population. The authors utilised a large cohort from the UK Biobank to investigate this important relationship, providing valuable insights. However, we would like to highlight a few key limitations and suggest future research directions that could strengthen the evidence in this area.

Firstly, the authors relied on self‐reported physical activity data, which is subject to potential recall bias and social desirability bias. Individuals may overreport or underreport their physical activity levels, leading to misclassification and potentially biassing the observed associations [2]. The use of objective measures, such as accelerometers or activity trackers, could provide more accurate and reliable assessments of physical activity, reducing the risk of measurement error. Indeed, through UK Biobank, several studies have reported high quality research on accelerometer‐measured physical activity and disease prognosis [3, 4].

Secondly, the authors' adjustment for covariates did not include dietary factors, which are known to have a significant impact on kidney function. Dietary intake of protein, sodium and other nutrients can influence serum creatinine and cystatin C levels, potentially confounding the relationship between physical activity and estimated glomerular filtration rate (eGFR) [5, 6]. Future studies should consider incorporating detailed dietary information, such as nutrient intake and dietary patterns, to better understand the complex interplay between physical activity, diet and kidney health.

Additionally, the authors focused their analysis on the general population, which may have different characteristics and risk profiles compared to individuals with pre‐existing chronic kidney disease (CKD) or other comorbidities. It would be valuable to conduct subgroup analyses or stratified models to explore the potential differential effects of physical activity changes on kidney function in specific patient populations, such as those with CKD, diabetes or cardiovascular disease. This approach could provide more targeted insights and guide the development of tailored physical activity recommendations for individuals at higher risk of kidney dysfunction.

Furthermore, the authors utilised eGFR as the primary outcome, which is an estimated measure of kidney function. While eGFR is widely used in clinical practice, it may not accurately reflect true glomerular filtration rate, especially in the context of changing muscle mass and body composition associated with physical activity [7]. Future studies could consider incorporating direct measures of kidney function, such as iohexol or inulin clearance, to provide a more precise assessment of the relationship between physical activity and actual kidney function.

Finally, the authors' analysis was limited to two time points, which may not capture the dynamic nature of physical activity and its long‐term impact on kidney health. Longitudinal studies with repeated assessments of physical activity and kidney function over an extended period could shed light on the trajectories of these variables and their interplay over time. This approach could help elucidate the causal mechanisms and identify critical time windows for interventions to preserve kidney function.

In conclusion, the authors' work provides valuable insights into the association between changes in physical activity and kidney function in the general population. However, addressing the limitations outlined above, such as the use of objective physical activity measures, incorporation of dietary factors, exploration of subgroup differences and the inclusion of direct kidney function assessments, could further strengthen the evidence and guide the development of targeted physical activity recommendations for kidney health.

## Data Availability

Not applicable.
